# Effectiveness of the influenza and Tdap vaccination educational module (InTroDuce-Programme) on knowledge and intention for antenatal vaccination: A cluster randomised controlled trial protocol among pregnant women in Malaysian primary care clinics

**DOI:** 10.1371/journal.pone.0344651

**Published:** 2026-03-12

**Authors:** Hung Chiun Lau, Zamberi Sekawi, Siew Mooi Ching, Norhasliza Abu Bakar, Rahmat Dapari, Siti Rohani Mohamed Alias, Nor Hazlin Talib, Izzah Hazwani Dzulkifli, Nadiah Md Alwi, Habibah Abdul Hamid, Hazwan Mat Din

**Affiliations:** 1 Department of Family Medicine, Faculty of Medicine and Health Sciences, Universiti Putra Malaysia, Serdang, Selangor, Malaysia; 2 Department of Medical Microbiology, Faculty of Medicine and Health Sciences, Universiti Putra Malaysia, Serdang, Selangor, Malaysia; 3 Clinical Research Unit, Hospital Sultan Abdul Aziz Shah, Universiti Putra Malaysia, Serdang, Selangor, Malaysia; 4 Department of Community Health, Faculty of Medicine and Health Sciences, Universiti Putra Malaysia, Serdang, Selangor, Malaysia; 5 Bandar Baru Bangi Health Clinic, Ministry of Health Malaysia, Selangor, Malaysia; 6 Batu 9 Health Clinic, Ministry of Health Malaysia, Selangor, Malaysia; 7 Kajang Health Clinic, Ministry of Health Malaysia, Selangor, Malaysia; 8 Balakong Health Clinic, Ministry of Health Malaysia, Selangor, Malaysia; 9 Department of Obstetrics and Gynaecology, Faculty of Medicine and Health Sciences, Universiti Putra Malaysia, Serdang, Selangor, Malaysia; 10 Malaysian Research Institute on Ageing (Myageing®), Universiti Putra Malaysia, Serdang, Selangor, Malaysia; University of Adelaide School of Medical Sciences: The University of Adelaide Adelaide Medical School, AUSTRALIA

## Abstract

**Background:**

Vaccination during pregnancy represents a critical public health strategy to safeguard both mothers and infants against infections such as influenza and pertussis. However, uptake remains suboptimal both globally and in Malaysia, with influenza vaccine coverage persistently low and limited awareness of Tdap recommendations. These gaps are of concern given the heightened risk of severe illness and adverse pregnancy outcomes among pregnant women, and the vulnerability of infants under two months to pertussis-related morbidity and mortality.

**Aims:**

This study aims to develop, implement, and evaluate the effectiveness of a theory-driven digital education module (InTroDuce-Programme) in improving maternal knowledge, attitudes, and intention to receive influenza and Tdap vaccines during pregnancy.

**Methods:**

This is a cluster randomised controlled trial to be conducted between 15 July 2025 and 14 July 2027 across four public primary care clinics in Hulu Langat, Malaysia. Two clinics will be randomly allocated to the intervention arm and two to the control arm. A total of approximately 351 pregnant women aged 18 years or older and up to 27 weeks’ gestation will be recruited. The intervention group will receive the InTroDuce-Programme, a web-based video module in Bahasa Melayu, delivered during antenatal visits, while the control group will receive standard care. Data will be collected at baseline, immediately post-intervention (intervention group only), and one-month follow-up using a validated questionnaire. Primary outcomes are changes in vaccination knowledge and intention to vaccinate. Secondary outcomes include attitudes, perceived barriers, and associations with sociodemographic and clinical factors. Data will be analysed using descriptive statistics and Generalised Estimating Equations to account for cluster effects and repeated measures.

**Conclusions:**

This study will provide context-specific evidence on whether a digital, culturally tailored, Health Belief Model-based intervention can improve maternal vaccination literacy and intention in Malaysia. If effective, the InTroDuce-Programme could be integrated into routine antenatal care and inform broader maternal immunisation strategies in similar middle-income settings.

**Trial registration:**

ClinicalTrials.gov NCT06815250 dated 7th February 2025.

## Introduction

Vaccination during pregnancy is a crucial public health measure that protects both the mother and her unborn child from severe infectious diseases such as influenza and pertussis [1]. Despite robust evidence for their safety and efficacy, vaccination rates for influenza and pertussis during pregnancy remain suboptimal globally, with particularly low awareness and coverage in many low- and middle-income countries [2]. In Malaysia, a 2017 study reported that only 17.3% of pregnant women received the influenza vaccine [3], while an Immunise4Life survey found that awareness of the recommendation for Tdap and influenza vaccination during pregnancy was just 21.8% and 26.9%, respectively [4].

This gap in preventive care is concerning, as pregnant women face heightened vulnerability to respiratory infections due to physiological and immunological changes. Recent systematic reviews indicate that pregnant women are approximately four times more likely to be hospitalised with influenza compared to non-pregnant women [5]. In middle-income countries, the incidence of influenza is estimated at 88.7 cases per 10,000 pregnant women-months [6], with complications ranging from maternal hospitalisation and late pregnancy loss to reduced infant birthweight [6]. Maternal respiratory infection is also associated with adverse outcomes such as preterm birth and fetal growth restriction [7]. In Malaysia, pertussis continues to cause substantial morbidity and mortality, with the highest hospitalisation and fatality rates occurring among infants younger than two months who are not yet eligible for vaccination [8]. Nearly 90% of confirmed pertussis cases have occurred in under-vaccinated or unvaccinated infants under six months, leading to avoidable illness, parental workday losses, and additional healthcare costs [8].

The Malaysian experience highlights these risks. During the 2009 H1N1 pandemic, more than 12,000 influenza cases and 77 deaths were reported within six weeks [9]. At the peak of the 2023 outbreak, national surveillance data from the general population indicated that 23.7% of tested specimens were positive for influenza, underscoring the ongoing burden of seasonal epidemics. While pregnancy-specific national surveillance data are limited, pregnant women are recognised as a high-risk group with higher risks of severe illness, hospitalisation, and adverse pregnancy outcomes. Importantly, maternal influenza vaccination not only protects mothers but also reduces infant infections by up to 63% and respiratory illness by 29–36% [4]. In response to this burden, the Ministry of Health Malaysia recommends routine influenza and tetanus-diphtheria-acellular pertussis (Tdap) vaccination during pregnancy to reduce maternal complications and allow passive transfer of protective antibodies to infants [10]. Professional guidance from the 2024 Obstetrical and Gynaecological Society of Malaysia (OGSM) Maternal Immunisation Consensus Guidelines recommends Tdap vaccination during each pregnancy between 16 and 36 weeks of gestation, and inactivated influenza vaccination in any trimester of pregnancy. In Malaysia, maternal immunisation is delivered through Ministry of Health (MOH) facilities as part of routine antenatal care. Maternal Tdap vaccination has been incorporated into the national maternal immunisation schedule from the second half of 2025 and is provided at no cost to Malaysian pregnant women attending MOH facilities, with routine administration prioritised between 28 and 32 weeks of gestation in line with national implementation policy. In contrast, although influenza vaccination is recommended during pregnancy, it remains optional and self-funded, which may contribute to variability in uptake.

Despite robust evidence for safety and effectiveness [11,12], uptake remains low. Reported barriers include concerns about vaccine safety, limited knowledge of benefits, fears of adverse effects on the mother or fetus, and low perceived disease severity. Conversely, facilitators include strong recommendations from trusted healthcare providers, convenient access to vaccination during antenatal visits, clear communication of safety and benefits, and social support from family and peers [5,13].

Recent events, including the death of a high-profile international figure from influenza complications and outbreaks reported in Japan, may have heightened public awareness and concern regarding influenza vaccination [14,15]. This surge in awareness underscores the urgent need for effective strategies to sustain vaccine literacy and translate concern into uptake, particularly among high-risk groups such as pregnant women. Digital educational interventions have shown promise in addressing vaccine hesitancy and improving vaccination behaviours. Evidence from systematic reviews indicates that video-based interventions are more effective than text messages in conveying health information and motivating behavioural change [16]. Recent reviews of maternal vaccination interventions further demonstrate that Health Belief Model (HBM)–informed educational approaches, including multimedia and video-based formats, have been applied in antenatal settings to improve vaccination-related knowledge, attitudes, and intention, providing precedent for this intervention strategy [17]. The HBM, a well-established framework for explaining health-related behaviours, is especially suited for guiding intervention design by targeting constructs such as perceived susceptibility, severity, benefits, barriers, and self-efficacy [18].

Building on this evidence, we propose the Influenza and Tdap Vaccination Educational and Learning Module (InTroDuce-Programme), a web-based educational video developed using the Health Belief Model. The module is designed to improve maternal knowledge, address safety concerns and misconceptions, and ultimately increase uptake of maternal influenza and Tdap vaccination in Malaysia. A video-based format was selected to allow standardised delivery of complex and culturally sensitive messages—such as maternal and infant risk, vaccine safety, and religious considerations—in a time-efficient and engaging manner. High smartphone access among Malaysian adults supports the feasibility of this approach in routine antenatal care settings [19]. Compared with SMS-based messaging, mobile applications, or in-person counselling, a short web-based video is logistically feasible in busy Malaysian antenatal clinics and can be delivered during routine waiting periods without increasing healthcare provider workload. By delivering culturally tailored, evidence-based content in the national language, Bahasa Melayu, through accessible digital platforms, this intervention aims to overcome common barriers such as low awareness, misinformation, and limited counselling time during routine antenatal care. This study, therefore, aims to evaluate the effectiveness of the InTroDuce-Programme in improving maternal knowledge, attitudes, and future intention to receive influenza and Tdap vaccination during pregnancy.

## Materials and methods

### Study type and design

This study is a cluster randomised controlled trial, with randomisation conducted at the clinic level to minimise contamination between participants. It will be implemented in four public primary care clinics in the Hulu Langat district. Clinics will first be categorised by setting (urban or suburban), and randomisation will be conducted within each category using a computer-generated sequence. Two clinics (one urban and one suburban) will be allocated to the intervention arm, where participants will receive the InTroDuce-Programme, and the remaining two clinics will serve as controls, providing standard antenatal care.

### Study duration

The study duration is 24 months, from 15 July 2025 to 14 July 2027. The initial phase will involve research planning and development of the web-based educational module (InTroDuce-Programme), followed by trial implementation and effectiveness evaluation. Recruitment is anticipated to commence on 2 February 2026 and is expected to continue for approximately six months, until August 2026, subject to administrative and logistical approvals. At enrolment, baseline data (T0) will be collected for both intervention and control groups. Participants allocated to the intervention group will receive the educational module immediately after completion of the baseline assessment (T0) and will complete an immediate post-intervention assessment (T1) to evaluate short-term knowledge acquisition. A follow-up assessment at one-month post-intervention (T2) will be conducted for both intervention and control groups.

**Fig 1:**
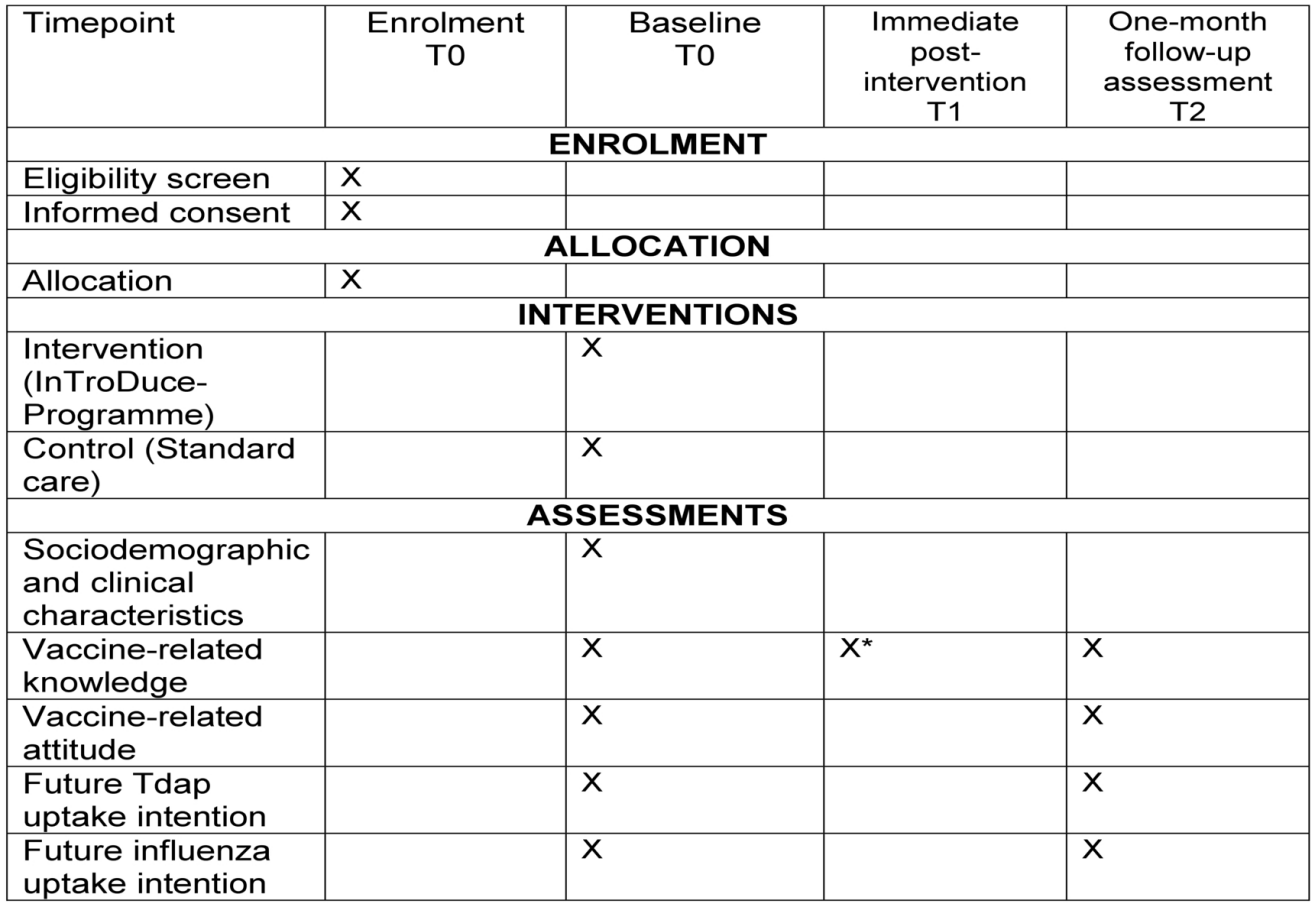
Schedule of enrolment, intervention and assessment. * Immediate post-intervention assessment conducted in the intervention group only. T0 = Baseline (pre-intervention); T1 = Immediate post-intervention assessment (intervention group only); T2 = One-month follow-up assessment. ‘X’ indicates when the activity or assessment occurs.

Data collection is expected to be completed by August 2026, with results anticipated by mid-2027. The schedule of enrolment, intervention, and assessments is outlined in Fig 1.

### Study location

The study will take place in four primary care clinics in Hulu Langat district, Selangor, Malaysia. Kajang Health Clinic and Bandar Baru Bangi Health Clinic represent urban sites, while Batu 9 Health Clinic and Balakong Health Clinic represent suburban sites. These were chosen to ensure a mix of geographic and demographic representation.

### Study population

The study population consists of pregnant women attending the four participating clinics during the recruitment period.

### Inclusion and exclusion criteria

**Inclusion criteria:**

1. Pregnant women aged 18 years and older.
2. At or before 27 weeks of gestation, ensuring that participants are recruited prior to the recommended vaccination window.
3. Able to understand and provide informed consent.

**Exclusion criteria:**

1. History of severe anaphylactic or allergic reactions to previous influenza or Tdap vaccination.
2. Non-Malaysian citizens

### Sample size estimation

Sample size was calculated using OpenEpi software, based on O’Leary et al. (2019), which reported a 19% absolute difference in vaccine intention between intervention (55%) and control groups (36%) [20]. To detect this difference with 80% power and a 5% two-sided significance level, a total of 216 participants were required for an individually randomised design. As this is a cluster randomised trial, the sample size was adjusted for clustering using a design effect of 1.3, assuming an intracluster correlation coefficient (ICC) of 0.05. This ICC value was selected based on published cluster trials of educational and behavioural interventions in primary care, where ICCs for knowledge and intention outcomes are typically low (<0.05) [21]. Given the limited number of clusters (four clinics), the sample size supports a pragmatic, early-phase evaluation, and is not powered for definitive effectiveness conclusions. Findings are intended to inform the design of a future, fully powered trial. Allowing for 20% attrition, the final sample size is 351 participants (88 per clinic).

### Sampling method and participant recruitment

Cluster randomisation will occur at the clinic level. Within each clinic, consecutive sampling of eligible pregnant women will be used until the target sample size is achieved. Research officers will screen women during antenatal visits, obtain informed consent, and enrol participants.

#### Randomisation.

To minimise contamination, randomisation will be conducted at the clinic (cluster) level rather than the individual level. This approach reduces the risk of participants within the same clinic sharing information or influencing each other’s behaviour. Entire clinics will therefore be assigned to either the intervention or control arm, ensuring that participants in each site receive consistent exposure to either the InTroDuce-Programme or standard care.

- **Intervention clinics:** Pregnant women will receive the InTroDuce-Programme, a web-based educational module on influenza and Tdap vaccination.
- **Control clinics:** Pregnant women will receive standard antenatal care in accordance with Malaysian Ministry of Health guidelines, including routine antenatal follow-up and usual verbal counselling on Tdap vaccination. Counselling on influenza vaccination and standardised printed or digital maternal vaccination materials are not routinely or systematically provided.

#### Sequence generation and allocation concealment.

Participating clinics will be randomly allocated to intervention or control arms using a computer-generated random sequence. Randomisation will be performed by an independent researcher who has no role in recruitment or data collection. This process ensures allocation concealment, as investigators enrolling participants will not influence or have prior knowledge of the clinic assignments, thereby minimising selection bias.

#### Blinding.

Blinding of participants and providers is not feasible due to the nature of the intervention: women in the intervention group will receive a digital educational module and will therefore be aware of their allocation. To minimise contamination, randomisation is conducted at the clinic level, and the selected intervention and control clinics are located at least 10 kilometres apart to reduce the likelihood of cross-participant interaction. While participants and healthcare providers will be aware of group assignment, outcome assessors and data analysts will remain blinded to allocation through the use of anonymised, coded datasets, thereby minimising assessment bias.

### Research phases and tools

This protocol outlines the development, implementation, and evaluation of the InTroDuce-Programme, a web-based educational intervention designed to improve knowledge and future intention for influenza and Tdap vaccination among pregnant women. The study consists of two phases.

#### Phase 1: Development of the educational video module using the Health Belief Model (HBM).

The InTroDuce-Programme will be developed as a short, web-based video module (~7 minutes) to promote maternal vaccination. Its design will be guided by the HBM, targeting constructs such as perceived susceptibility, severity, benefits, barriers, and self-efficacy. To increase behavioural impact, the module will integrate simplified and empathetic messaging, visual storytelling, and culturally relevant content in Bahasa Melayu. Local issues, including vaccine myths (halal status), will be addressed in line with the Malaysian Maternal Immunisation Consensus Guidelines [22].

To address perceived barriers, particularly concerns related to vaccine safety and religious permissibility, the module includes messages such as: “Influenza and Tdap vaccines given during pregnancy are inactivated or subunit vaccines that do not contain live pathogens and are safe for both the mother and the baby.” In addition, the module states that “the use of influenza and Tdap vaccines during pregnancy is considered harus (permissible under Islamic law) when they are proven to be safe, contain no impermissible substances that remain, or when the original substances have undergone istihalah (complete chemical transformation), rendering the final product suci (ritually pure), and are used to protect life.” Clarification of “harus” status is relevant in the Malaysian context, where religious permissibility under Islamic jurisprudence may influence vaccine acceptance among pregnant women.

Message framing is differentiated according to maternal risk perception. Messages on pertussis (Tdap) emphasise protection of the newborn, reflecting the common perception that pertussis primarily threatens infants, particularly in early life. In contrast, influenza messages highlight protection of both the mother and the baby, acknowledging influenza as a health risk mainly perceived by pregnant women to affect themselves. This differentiated framing aligns message emphasis with maternal concerns and relevant HBM constructs to enhance message relevance and acceptability.

The development process will include:

1. **Content and theory development**: establishing key educational messages guided by the HBM.
2. **Module drafting**: preparing initial materials (text, audio, and visual) addressing all six HBM constructs.
3. **Expert review and content validation**: input from family medicine specialists, obstetricians, and public health experts to ensure accuracy and relevance.
4. **Refinement**: revisions based on expert feedback.
5. **Pilot testing**: evaluation with a small group of pregnant women (n = 20) will be conducted to assess feasibility and study flow. Face validation will be conducted with a subset of participants using a structured face validation form to assess clarity, user-friendliness, and engagement. Quantitative ratings and qualitative feedback will be reviewed by the research team, and identified issues will be addressed through revisions to content, visuals, and delivery before full trial rollout. Participants involved in pilot testing will not be included in the main trial.
6. **Final refinement**: adjustments based on pilot testing before full trial implementation.

The module will be deployed via a password-protected website accessible on smartphones.

**Note:** The InTroDuce-Programme explicitly incorporates HBM constructs to address key determinants of vaccination behaviour. Each module component aligns with a specific HBM construct to improve knowledge, address perceived barriers, and empower informed decision-making among pregnant women (see Table 1).

**Table 1:** Mapping of health belief model constructs to the InTroDuce-Programme components.

Construct	I. Risk of influenza & pertussis	II. Vaccination benefits	III. Address concerns	IV. Provide triggers	V. Empower decision-making
Perceived Susceptibility	Statistics on maternal/ infant morbidity/ mortality due to infections				
Perceived Severity	Explanation of suppressed immune system and infection risks				
Perceived Benefits		Evidence of vaccine effectiveness; maternal antibody protection			
Perceived Barriers			Safety data, myths vs facts, guideline recommendations		
Cues to Action				Vaccination recommendations from healthcare providers	
Self-Efficacy					Self-assessment quiz; vaccination information and access; talk to provider

#### Phase 2: Implementation and effectiveness measurement.

This phase evaluates the effectiveness of the InTroDuce-Programme through a cluster randomised controlled trial.

**Study design**: Four primary care clinics (two urban, two suburban) will participate. Clinics will first be categorised by setting (urban or suburban), and randomisation will be conducted within each category using a computer-generated sequence. One clinic in each category will be randomly assigned to the intervention arm (InTroDuce-Programme) and one to the control arm (standard antenatal care). Randomisation at the clinic level minimises contamination, and participating sites are at least 10 kilometres apart.

**Intervention (InTroDuce-Programme):** Pregnant women randomised to the intervention arm will be offered the **InTroDuce-Programme**, a web-based video module hosted on a subscription-only research website. The content is presented in Bahasa Melayu with culturally appropriate visuals and includes statistics on maternal and infant morbidity and mortality, derived from published international literature, evidence of vaccine effectiveness, safety data, local guideline recommendations and self-assessment prompts. The content and structure of the module are guided by the Health Belief Model, as described in the Intervention Development section (Table 1). Participants will view the module on their own smartphone during their antenatal visit immediately after baseline assessment (T0), either on their own smartphone or on a device provided on-site by the research assistant when personal smartphone access or mobile data is unavailable. A research assistant will provide a brief introduction, assist with login and remain available to answer technical questions. Encouragement to complete viewing will be limited to procedural reminders to ensure full exposure to the intervention, and no additional counselling or persuasive discussion will be provided. A standardised script will be used across sites to maintain consistency. This facilitation is implemented to ensure intervention fidelity within the trial setting. Participants may re-watch the video at the one-month follow-up.

**Control condition:** Women in the control arm will receive routine antenatal care as currently practiced in participating clinics, in accordance with Ministry of Health guidelines. This includes general health education on diet, exercise, and pregnancy-related topics. As part of usual practice, verbal counselling on tetanus–diphtheria–pertussis (Tdap) vaccination is provided during antenatal care. In contrast, influenza vaccination is not included in the National Immunisation Programme, and counselling regarding maternal influenza vaccination is not routinely or systematically provided. No standardised printed or digital educational materials on maternal influenza or Tdap vaccination are routinely provided. This approach is ethically appropriate, as no recommended care is withheld, and it enables evaluation of the added value of the InTroDuce-Programme under real-world conditions. Participants in the control arm will not have access to the InTroDuce-Programme during the study, and no restrictions will be placed on other sources of information or vaccine counselling they may receive.

**Adherence and fidelity:** Completion of the intervention will be monitored by research assistants. Research assistants will document whether participants watch the module in full during the clinic visit. A reminder will be sent after one week only to participants who did not complete viewing of the module during the clinic visit. Any issues (e.g., internet access problems) will be recorded. Clinic staff will receive a briefing session to ensure consistent delivery across sites.

**Discontinuation:** As the intervention is educational and carries minimal risk, there are no formal criteria for discontinuation beyond participant withdrawal of consent. Participants may stop watching the video at any time and remain in the study unless they withdraw entirely.

**Concomitant care:** Participants in both arms will continue to receive standard antenatal services and remain free to accept or decline influenza or Tdap vaccination through routine antenatal care pathways. Vaccinations were not funded or provided by the study. Those in the intervention group will be asked not to share the module with participants from control clinics during the study, but no restrictions will be placed on access to other information sources.

This phase will provide context-specific evidence on the effectiveness of a digital educational intervention in improving vaccine literacy and intention among pregnant women in Malaysia.

### Data collection and instruments

**Fig 2:**
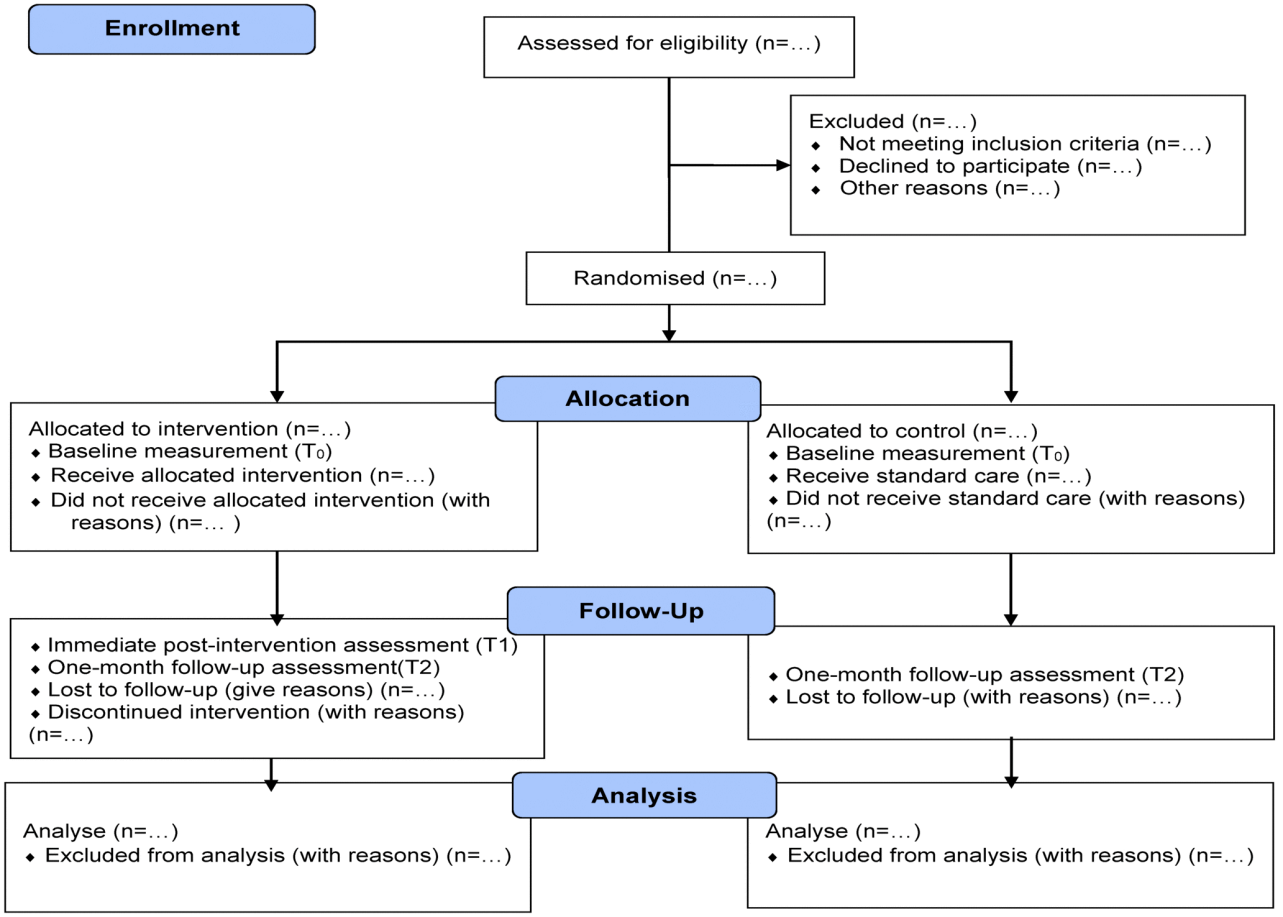
CONSORT flow diagram of enrolment, allocation, follow-up and analysis.

Data will be collected at baseline (T0) and one-month follow-up (T2) for both intervention and control groups. Upon obtaining consent, the research officer will administer a baseline questionnaire via Google Form to collect sociodemographic and clinical data, knowledge, attitudes and vaccination intention. Participants in the intervention arm will then watch the video module during the same visit and will complete an additional immediate post-intervention assessment (T1), which will assess short-term knowledge acquisition. Both intervention and control participants will complete the follow-up questionnaire at T2. Fig 2 illustrates the CONSORT flow diagram for enrolment, allocation, follow-up and analysis.

#### Questionnaire.

The questionnaire will consist of 13 knowledge items, six attitude items and two intention items. It includes sections on screening and eligibility questions, sociodemographic and clinical variables, knowledge about vaccination during pregnancy, knowledge of pertussis and influenza infection, attitudes towards vaccination, and intention to receive influenza and Tdap vaccines. Items will be presented in multiple-choice, True/False/Don’t know, and rating scale formats. Questions will be adapted, with contextual modifications, from validated tools published by Hong et al. (2023) in the *Singapore Medical Journal*, available under the Creative Commons Attribution-NonCommercial-ShareAlike 4.0 License [23].

#### Validation of questionnaire.

Content validity of the adapted questionnaires will be conducted among six subject matter experts, which will include family medicine specialists, obstetricians and public health specialists. Face validation among a group of pregnant women will be conducted to ensure clarity, relevance, and comprehensibility of the adapted questionnaire, allowing refinement according to their feedback. The overall validity and reliability of the tool will be supported through both expert input and pilot testing. Internal consistency reliability (Cronbach’s alpha) will be assessed separately for the knowledge and attitude scales. The intention scale consists of two items and will therefore be analysed as a composite outcome.

### Outcomes

**Primary outcomes:**

1. Vaccination knowledge: Change in knowledge score from T0 to T2 based on correct responses to the knowledge items.
2. Vaccination intention: Change in intention to receive influenza and Tdap vaccination during the current pregnancy from T0 to T2, measured via two items (Yes/No/Not sure) coded ordinally (No = 0, Not sure = 1, Yes = 2).

**Secondary outcomes:**

3. Attitudes and perceived barriers: Scores on the six attitude items capturing constructs such as perceived susceptibility, severity, benefits, barriers, cues to action and self-efficacy.
4. Factors associated with lower knowledge or intention: Associations between sociodemographic and clinical variables (e.g., age, parity, education, household income) and outcomes.
5. Preferences for information sources and modes: Responses on preferred sources/modes of vaccine information (e.g., healthcare provider, WhatsApp, authorised website) to inform communication strategies.

### Data analysis

Data will be analysed using IBM SPSS Statistics version 29. Participant characteristics will be described using descriptive statistics at both the individual participant level and the cluster (clinic) level, with categorical variables expressed as frequencies and percentages, and continuous variables summarised as means with standard deviations or medians with interquartile ranges, as appropriate. Missing data will be handled using complete-case analysis, with planned sensitivity analyses specified in the Statistical Analysis Plan. Given the small number of clusters (k = 4), the primary analysis will be conducted at the cluster level. For each clinic, summary measures will be calculated for the primary outcomes, including the mean change in knowledge score and the proportion of participants reporting positive vaccination intention. The intervention effect will be assessed by comparing these clinic-level summaries between intervention and control clinics using independent samples t-tests, which are intended as exploratory comparisons given the limited number of clusters and will be interpreted cautiously, with consideration of effect sizes and confidence intervals in addition to statistical significance.

Secondary exploratory analyses will be conducted at the individual level using Generalised Estimating Equations (GEE) with an exchangeable correlation structure to account for clustering and repeated measures. For continuous outcomes (knowledge scores), an identity link will be used; for ordinal intention scores, a cumulative logit link will be applied. Models will adjust for baseline values and prespecified covariates such as age and education. Findings from these analyses will be interpreted cautiously given the limited number of clusters. Exploratory logistic regression analyses will be conducted to examine factors associated with low vaccination intention at individual level. These analyses are intended to be descriptive and hypothesis-generating and will be interpreted cautiously in light of the small number of clusters.

Additional sensitivity analyses will be conducted to assess model stability, including comparison of unadjusted and covariate-adjusted models and consistency between cluster-level and individual-level exploratory analyses. Statistical inference will be interpreted in the context of the exploratory nature of the analyses, with emphasis placed on effect sizes and confidence intervals. A two-tailed p-value < 0.05 will denote statistical significance.

A detailed Statistical Analysis Plan will be finalised prior to outcome analysis and before access to outcome data. Reporting will follow the CONSORT 2010 extension for cluster randomised trials.

### Data management

Data will be collected directly through Google Forms, which automatically records responses into a secure, password-protected Google Sheet. Built-in validation rules (e.g., mandatory fields, age limits) will minimise missing or out-of-range values. The research team will conduct regular quality checks to identify and correct inconsistencies. All study data will be stored in accordance with institutional and national data protection requirements on encrypted institutional servers, accessible only to authorised members of the research team. Personal identifiers (e.g., names and contact details) will not be included in the main survey dataset. Each participant will instead be assigned a unique study identification number, with the linkage file containing personal identifiers stored separately under restricted access. Data will be retained for seven years after study completion, in line with institutional policy, and will then be permanently deleted. De-identified datasets and analysis codes may be made available to qualified researchers upon reasonable request and approval from the relevant ethics committee and data custodians.

### Ethics approval, trial registration, and consent to participate

The study received ethical approval from the Medical Research and Ethics Committee, Ministry of Health Malaysia, through the National Medical Research Registry (NMRR ID: 25-00625-ECZ (IIR)); approval date: 22 April 2025. The approval is valid until 21 April 2026, and an extension will be requested if required. The protocol approved by the Medical Research and Ethics Committee is provided in S1 Protocol. It is registered with ClinicalTrials.gov (NCT06815250; 7 February 2025). Permission will be obtained from the state health department and district office before data collection. The trial will be conducted in accordance with the Declaration of Helsinki and the Malaysian Guidelines for Good Clinical Practice. Any important protocol amendments will be submitted for prior approval to the ethics committee and updated in the trial registry. Relevant changes will also be communicated to investigators, reported in the final manuscript, and disclosed to journal editors at submission. Participants will be informed about the study during routine clinic visits. Written informed consent will be obtained from all participants prior to enrolment and will be documented electronically. The consent process will provide participants with the full information sheet and require active acknowledgement before submission. No minors will be included. A token of appreciation (Ringgit Malaysia 30) will be provided upon completion of the study. The patient information sheet and informed consent form are provided in S1 Appendix.

### Safety considerations

The intervention involves only an educational module and questionnaires, posing minimal risk. Potential discomfort is limited to the time spent answering questions. Participants may withdraw at any time without impact on their usual care.

### Privacy and confidentiality

Personal identifiers will be kept in a separate, password-protected file accessible only to the principal investigator and data manager. Only study IDs will appear on questionnaires and datasets. No personal information will be included in reports or publications.

The complete SPIRIT checklist is provided in S1 Checklist.

## Discussion

This protocol describes the systematic development and planned evaluation of the InTroDuce-Programme, a theory-driven, web-based educational module to improve maternal knowledge, attitudes, and intention to receive influenza and Tdap vaccination during pregnancy. Grounded in the HBM, the intervention directly addresses common barriers such as low awareness, safety concerns, and cultural misconceptions, while strengthening facilitators, including provider recommendation and informed decision-making.

The use of a cluster randomised controlled trial across primary care clinics will generate context-specific evidence on whether a digital, culturally tailored intervention can effectively improve vaccination literacy and intention among pregnant women in Malaysia. In addition to the primary outcomes, secondary analyses will explore attitudinal shifts, perceived barriers, and sociodemographic predictors of low uptake intention, thereby enriching the understanding of maternal vaccination behaviours in a middle-income country setting.

If effective, the InTroDuce-Programme can be scaled and integrated into existing antenatal services, including clinic waiting areas and antenatal classes, thereby supporting ongoing national maternal immunisation initiatives. The web-based, mobile-accessible design aligns with Malaysia’s digital-health strategies by enabling standardised, low-cost delivery of evidence-based vaccine education without increasing clinical workload. Integration of such digital educational tools may help address persistent gaps in vaccine awareness and acceptance within routine antenatal care. Beyond Malaysia, the findings may also inform maternal immunisation strategies in other low- and middle-income countries facing similar challenges of low vaccine awareness and uptake.

### Dissemination plans

Findings will be disseminated through national and international conferences and submissions to peer-reviewed journals on public health, vaccination, primary care, and maternal health. Key results will be shared with policymakers, maternal health programme managers, and frontline healthcare providers through summary reports and presentations to support evidence-based decision-making. The InTroDuce-Programme may also be adapted for practical use in antenatal classes and clinic waiting areas to extend its reach. The full protocol and analysis plan will accompany the final publication. De-identified data may be shared in line with data management procedures.

### Strength and limitations

This study is among the first in Malaysia to evaluate a web-based educational module on maternal vaccination, employing a cluster randomised controlled design to minimise contamination. Its digital format is scalable and feasible for integration into routine antenatal care. Analyses of barriers and sociodemographic factors will provide valuable insights to guide targeted interventions. Limitations include the lack of participant blinding, which may introduce response bias; the relatively short follow-up period, which may not capture long-term uptake; and reliance on self-reported intention rather than verified vaccination. This study primarily assessed changes in knowledge and behavioural intention rather than verified vaccine uptake. Although confirmed uptake would represent a more definitive measure of effectiveness, this was not feasible within the scope of the current protocol due to the timing of national Tdap implementation and resource constraints. It is acknowledged that willingness or intention to receive vaccination may not necessarily translate into actual vaccine uptake. Intention was therefore selected as a pragmatic and theoretically grounded outcome, and future studies should evaluate the impact of the intervention on confirmed vaccination uptake.

Intervention delivery involved monitoring by research assistants to ensure fidelity, which may not fully reflect routine clinical practice where such supervision is unlikely. The intervention was designed as a brief, standalone video, allowing flexibility for future dissemination through routine channels, such as display in healthcare facility waiting areas, although the effectiveness of such approaches in rural or low digital access settings was not evaluated in this study. In addition, the small number of clusters limits statistical efficiency and the precision of cluster-level effect estimates. As the primary cluster-level analyses are exploratory in nature, findings will be interpreted cautiously, with emphasis placed on effect sizes and confidence intervals rather than statistical significance alone. The intracluster correlation coefficient assumed for sample size estimation was based on published primary care trials, and ICC values may differ in Malaysian primary care settings, which could affect the precision of effect estimates. The sample size calculation was also informed by a single published study conducted in a high-income setting, which may limit the generalisability of the assumed effect size to the local context. Finally, some residual cross-clinic contamination is possible despite randomisation at the clinic level. Although clinics were located at least 10 km apart and participants in the intervention group were reminded not to share the module with others, sharing of digital content cannot be fully controlled during the study period.

### Termination of study

The study may be terminated at any time at the discretion of the investigators. If termination occurs, participants will be notified promptly, and follow-up arrangements will be made as appropriate.

## Supporting information

S1 Protocol Approved study protocol by the Medical Research and Ethics Committee, Ministry of Health Malaysia. (PDF)

S1 Appendix Patient information sheet and informed consent form. (DOCX)

S1 Checklist SPIRIT checklist. (PDF)
