# Supplementary material for: Effectiveness of the influenza and Tdap vaccination educational module (InTroDuce-Programme) on knowledge and intention for antenatal vaccination: A cluster randomised controlled trial protocol among pregnant women in Malaysian primary care clinics
Source: PLoS One. 2026 Mar 12;21(3):e0344651. doi: 10.1371/journal.pone.0344651 (PMC12981446; doi:10.1371/journal.pone.0344651)
Supplement: S1 Protocol — (PDF) [file pone.0344651.s001.pdf]

Study Protocol
Effectiveness of the Influenza and Tdap Vaccination Educational and Learning Module (Introduce-Programme) in Improving Knowledge and Future Uptake of Vaccination among Pregnant Mothers in Primary Care Clinics: A Randomised Control Trial

Protocol number, version number and date:
Protocol number – InTroDuce Programme 1, Version 1.0, 22 Januari 2025

Name and Institution of Principal investigator:

Dr Lau Hung Chiun, Jabatan Perubatan Keluarga, Fakulti Perubatan dan Sains Kesihatan, Universiti Putra Malaysia

Name and Institution of Co-Investigators:
1. Professor Dr. Ching Siew Mooi, Jabatan Perubatan Keluarga, Fakulti Perubatan dan Sains Kesihatan, Universiti Putra Malaysia (Co-investigator at Klinik Kesihatan Batu 9)
2. Dr Nor Hazlin Binti Talib, Klinik Kesihatan Batu 9
3. Dr Nur Harnani, Klinik Kesihatan Kajang
4. Dr Nadiah Binti Md Alwi, Klinik Kesihatan Balakong
5. Dr Siti Rohani bt. Mohamed Alias, Klinik Kesihatan Bangi

Name and address of Sponsor:
Universiti Putra Malaysia, Jalan Universiti 1, 43400 Serdang, Selangor

Study site/s:
Klinik Kesihatan Kajang
Klinik Kesihatan Bangi
Klinik Kesihatan Batu 9
Klinik Kesihatan Balakong

Contents

List of Abbreviations 3
Research Synopsis 4
1.0 Background and Significance 6
2.0 Objective 7
3.0 Methodology 7
3.1 Study Type and Design 7
3.2 Participants 9
3.3 Study Population 10
3.3.1 Inclusion Criteria 10
3.3.2 Exclusion Criteria 10
3.3.3 Withdrawal Criteria 10
3.4 Sample Size 11
3.5 Research phases and tools 11
3.6 Data Collection 13
3.7 Study Duration and Timeline 14
3.8 Study Visits and Procedures 15
3.9 Statistical Analysis Plan 15
3.10 Risk and benefit to study participants 15
3.11 Risk Benefit Assessment 17
3.12 Ethics of Study 17
3.13 Informed Consent/Assent Process 17
3.14 Privacy and Confidentiality 17
3.15 Conflict of Interest 18
3.16 Publication Policy 18
3.17 Termination of Study 18
3.18 References 18

List of Abbreviations

Tdap	Tetanus diphtheria, acellular pertussis
RCT	Randomised controlled trial
KK	Klinik Kesihatan

Research Synopsis

Study title	Effectiveness of the Influenza and Tdap Vaccination Educational and Learning Module (Introduce-Programme) in Improving Knowledge and Future Uptake of Vaccination among Pregnant Mothers in Primary Care Clinics: A Randomised Control Trial
Study Population	All pregnant women attending Klinik Kesihatan Kajang, Klinik Kesihatan Bangi, Klinik Kesihatan Batu 9, and Klinik Kesihatan Balakong during the period of 1st May 2025- 30th April 2026
Study Design	A randomised controlled trial (RCT) will be conducted in four primary care clinics in Hulu Langat district which are Klinik Kesihatan Kajang, Klinik Kesihatan Bangi, Klinik Kesihatan Batu 9 and Klinik Kesihatan Balakong. The study duration is 12 months, with recruitment over 6 months. A second follow-up will be conducted one month after providing the InTroDuce-Programme. Two clinics will be randomly assigned to administer the InTroDuce-Programme, while the other two will be provided with standard care, in order to minimise contamination.
General Objective	To evaluate the effectiveness of the InTroDuce-Programme in improving knowledge and future uptake of influenza and Tdap vaccination among pregnant mothers.
Specific Objectives	1) To determine the sociodemographic factors and clinical variables among study population.<br>2) To determine the prevalence of future vaccination uptake following the InTroDuce-Programme.<br>3) To evaluate the level of participants' attitudes and barriers towards future vaccination uptake before and after the InTroDuce-Programme.<br>4) To determine the effectiveness of the InTroDuce-Programme in improving knowledge and future uptake of influenza and Tdap vaccination among pregnant mothers.<br>5) To determine the factors associated with low future uptake of vaccine with socio-demographic factors (age, race, income, education) among pregnant mothers.
Study endpoints/outcomes	**Primary Outcome**: Improvement in participants' knowledge of vaccination safety, benefits, and future uptake intentions.<br>**Secondary Outcomes**: Identification of barriers to vaccination uptake and socio-demographic factors influencing outcomes.

Sample Size	351 participants
Study Duration	1st October 2024- 31st December 2026

1.0 Background and Significance

Vaccination during pregnancy is a crucial public health measure that prevent both the mother and her expectant child from getting severe infectious diseases such as influenza and pertussis (1). However, despite strong evidence supporting their safety and effectiveness, vaccination rates towards influenza (2), pertussis (3) during pregnancy remain suboptimal globally. This gap in preventive care is exacerbated in middle income country like Malaysia and presents a significant public health challenge that requires innovative, evidence-based solutions. Addressing this gap is particularly important as pregnant women face heightened vulnerability to respiratory infections due to physiological and immunological changes during pregnancy. Recent systematic reviews indicate that pregnant women are approximately four times more likely to be hospitalized with influenza compared to non-pregnant women (4).

The incidence of influenza, adjusted for the population of women of childbearing age in middle-income countries were 88.7 cases per 10,000 pregnant woman-months during the influenza season(5) . Complications can be severe, including increased risks of hospitalization, late pregnancy loss, and reduced infant birthweight (5). Furthermore, maternal infection with respiratory viruses has been associated with adverse pregnancy outcomes, including preterm birth and fetal growth restriction (6). Similarly, pertussis poses a substantial threat to infants under two months of age, who experience the highest rates of hospitalization and death before completing their primary vaccination schedule (7).

In Malaysia, the Ministry of Health Malaysia has issued recommendations for routine influenza and tetanus-diphtheria-acellular pertussis (Tdap) vaccination during pregnancy to mitigate the risks (8). Despite these vaccines have demonstrated robust safety profiles and effectiveness in protecting both mother and infant (9,10), the low vaccination uptake has been reported due to the presence of multiple barriers such as vaccine safety, inadequate provider recommendations, limited access to vaccination services, and socioeconomic disparities (11). Studies have shown that these barriers are particularly pronounced among socioeconomically disadvantaged populations (12).

Digital educational interventions have emerged a promising tool to address vaccine hesitancy and increase uptake rates. A systematic review reported that digital interventions using video appeared more effective than text messages in effectively deliver health information for behavioral changes (13).

Leveraging these findings, the Influenza and Tdap Vaccination Educational and Learning Module (InTroDuce-Programme) was developed as a comprehensive, web-based educational intervention to provide vaccine related information and to address concerns in order to improve maternal vaccination uptake in Malaysia. This research is particularly timely and relevant given the ongoing global emphasis on vaccine-preventable diseases and the need for evidence-based interventions to improve maternal vaccination rates in middle-income countries. By targeting pregnant women through digital-based education module, this intervention aims to improve maternal knowledge, address safety concerns and ultimately improving

maternal uptake vaccination rates in Malaysia.

2.0 Objective

General objective: To evaluate the effectiveness of the InTroDuce-Programme in improving knowledge and future uptake of influenza and Tdap vaccination among pregnant mothers.

Specific objectives:

1) To determine the sociodemographic factors and clinical variables among study population.
2) To determine the prevalence of future vaccination uptake following the InTroDuce-Programme.
3) To evaluate the level of participants' attitudes and barriers towards future vaccination uptake before and after the InTroDuce-Programme.
4) To determine the effectiveness of the InTroDuce-Programme in improving knowledge and future uptake of influenza and Tdap vaccination among pregnant mothers.
5) To determine the factors associated with low future uptake of vaccine with socio-demographic factors (age, race, income, education) among pregnant mothers.

3.0 Methodology

3.1 Study Type and Design

This randomised controlled trial (RCT) will be conducted in four primary care clinics in Hulu Langat district which are Klinik Kesihatan Kajang, Klinik Kesihatan Bangi, Klinik Kesihatan Batu 9 and Klinik Kesihatan Balakong. The study duration is 12 months, with recruitment over 6 months. A second follow-up will be conducted one month after providing the InTroDuce-Programme. Two clinics will be randomly assigned to administer the InTroDuce-Programme, while the other two will be provided with standard care, in order to minimise contamination.

(Appendix A: consort diagram)

Appendix A

CONSORT Flow Diagram

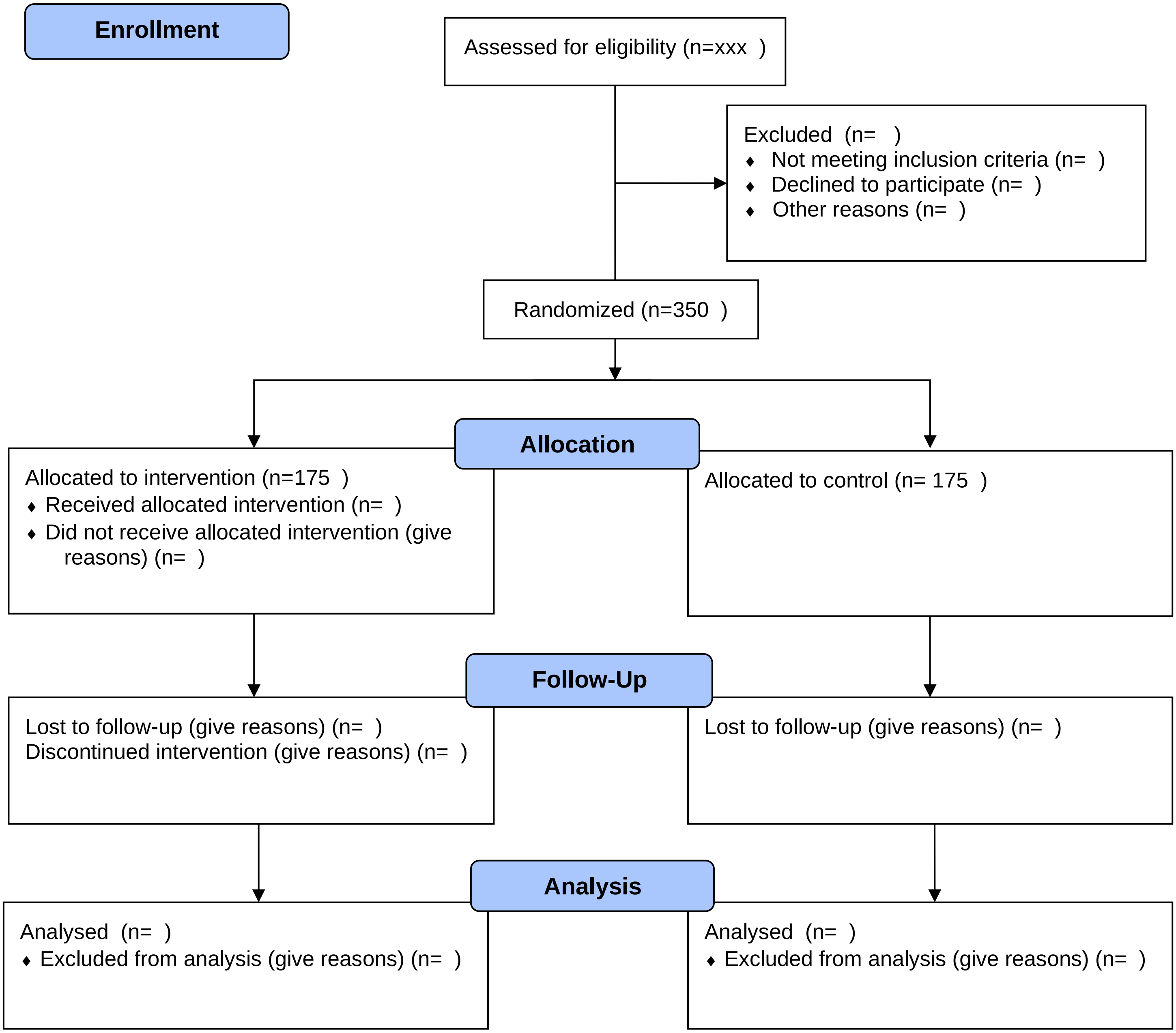

Cluster definition based on urban and suburban classification

Klinik Kesihatan Kajang and Klinik Kesihatan Bangi are classified as urban, while Klinik Kesihatan Batu 9 and Klinik Kesihatan Balakong are classified as suburban. These clinics were selected due to their geographic and demographic similarity, coupled with variability in socioeconomic, educational, cultural, and attitudinal factors. This selection ensures a balanced representation of diverse populations, enhancing the generalizability and applicability of the study findings. The approach is particularly valuable for comprehensively understanding the factors that influence vaccine knowledge and uptake among pregnant mothers.

Random Allocation of Clinics

- **Random Assignment**: One clinic from each category (urban and suburban) will be randomly assigned to either the intervention or control group using an online random number generator. This method ensures unbiased allocation and maintains the balance of geographic representation in both groups.
    - **Intervention Clinic**: The clinics will receive the **InTroDuce Programme**, where pregnant women attending the clinic will undergo the educational module on influenza and Tdap vaccination.
    - **Control Clinic**: The clinics will serve as the control group, where pregnant women will receive the standard antenatal care.

The rationale for Randomization is to minimise contamination. Randomizing at the clinic level (cluster randomization) rather than at the individual level will help reduce the risk of contamination, where participants in the same clinic may share information or influence each other's behaviour. By assigning entire clinics to either intervention or control, the study ensures that participants within each clinic receive consistent and isolated exposure to the educational materials or standard care.

3.2 Participants

- **Inclusion Criteria**:
    - Pregnant women aged 18 years and older
    - Before or equal to 32 weeks of gestation as we need to follow-up the patients again at 36 weeks of gestation before delivery occurs.
    - Able to understand and consent to participation in the study.
- **Exclusion Criteria**:
    - Pregnant women with severe anaphylactic or allergic reactions to previous dose of influenza or Tdap vaccination.
    - Non-Malaysian

Intervention

- **Intervention Group**: Participants will undergo the InTroDuce Programme, which includes an educational module delivered via website. The module will cover:
    - Importance of vaccination for both pregnant women and infant
    - Safety and efficacy of the influenza and Tdap vaccines.
    - Timing of vaccination.
    - Addressing common concerns and misconceptions.
- **Control Group**: Participants in the control group will receive the standard antenatal care

3.3 Study Population

All pregnant women attending Klinik Kesihatan Kajang, Klinik Kesihatan Bangi, Klinik Kesihatan Batu 9, and Klinik Kesihatan Balakong during the period of 1st May 2025-30th April 2026.

3.3.1 Inclusion Criteria

1. Pregnant women aged 18 years and older
2. Before or equal to 32 weeks of gestation as we need to follow-up the patients again at 36 weeks of gestation before delivery occurs.
3. Able to understand and consent to participation in the study.

3.3.2 Exclusion Criteria

1. Pregnant women with severe anaphylactic or allergic reactions to previous dose of influenza or Tdap vaccination.
2. Non-Malaysian

3.3.3 Withdrawal Criteria

Subjects can choose to withdraw at any time. Withdrawn subjects will not be replaced.

3.4 Sample Size

The sample size was calculated using openEpi software based on earlier study which demonstrated proportion of 55% in intervention group (website with vaccine information) and 36% in control group (usual care) (14) The final sample size is 351 based on the design effect of 1.3, 20% of non-response rate, 95% CI, 80% of power and significant value of 0.05.

Examples
Help

Sample Size:X-Sectional, Cohort, & Randomized Clinical Trials

Two-sided significance level(1-alpha):	95
Power(1-beta, % chance of detecting):	80
Ratio of sample size, Unexposed/Exposed:	1
Percent of Unexposed with Outcome:	36
Percent of Exposed with Outcome:	55
Odds Ratio:	2.2
Risk/Prevalence Ratio:	1.5
Risk/Prevalence difference:	19

	Kelsey	Fleiss	Fleiss with CC
Sample Size - Exposed	109	108	118
Sample Size-Nonexposed	109	108	118
Total sample size:	218	216	236

Design effect= 1.3

216x 1.3=281

Taking into consideration non-respondent rate of 20%, final sample size is 351.

3.5 Research phases and tools

This research protocol details the development, implementation, and evaluation of the InTroDuce-Programme, aimed at enhancing knowledge and increasing the future uptake of Influenza and Tdap vaccination among pregnant mothers. The study is divided into three sequential phases to ensure a comprehensive and systematic approach:

Phase 1: Needs Assessment

The first phase focuses on collecting essential information to guide the development of the intervention module, including:

1. **Literature Review**: A review on the existing research related to vaccine acceptance, hesitancy, and awareness among pregnant women to identify the knowledge gaps and factors associated with vaccine uptake will be conducted.
2. **Quantitative Study/Survey**: A baseline survey will be conducted to determine the target population's knowledge, attitudes, and agreement toward vaccine uptake.
3. **Guideline Review**: A detailed appraisal on the international and local vaccination guidelines with insights from the subject matter experts will be conducted to ensure the intervention align with evidence-based practices guidelines.

Phase 2: Development of the Interventional Educational Module

The second phase emphasizes the design and refinement of the educational module through an iterative process:

1. **Content and Theory Development**: The theoretical framework will be use in establishing key educational messages to ensure the module is well structured and impactful.
2. **Module Drafting:** Creating initial interventional materials, including text and visual components to address vaccine safety, efficacy, importance and common misconceptions related to vaccine.
3. **Expert Review and Content Validation**: Engaging subject matter experts like family medicine specialists, obstetricians and public health specialist to provide feedback and validate the scientific accuracy and relevance of the content.
4. **Refinement**: Revising the intervention educational module based on expert recommendations to enhance the clarity, accuracy and practicality.
5. **Method Selection**: Identifying practical methods for delivering the module (e.g., using WhatsApp as the digital platforms) and mechanisms for assessing its impact will be conducted using google form.
6. **Pilot Study**: Testing the module with a small group of pregnant women to identify practical and logistical challenges, as well as gathering participant feedback in terms of clarity of the questionnaire and the feasibility of the study.
7. **Module Evaluation**: A systematic evaluation based on Context, Input, Process, and Product (CIPP) of the educational model will be conducted.
8. **Final Refinement**: Incorporating feedback from the pilot study and module evaluation will be used to produce the finalized version of the intervention educational module.

Phase 3: Implementation and Effectiveness Measurement

The final phase involves the intervention implementation and evaluating its effectiveness through a randomised control trial (RCT):

1. **Study Design**: A cluster RCT will be used to compare the effectiveness of the **InTroDuce-Programme** (intervention group) versus standard care (control group) in improving the future vaccine uptake.
2. **Outcome Measurement**:
3. The module's effectiveness will be assessed using pre- and post-intervention questionnaires:
    1. **Primary Outcome**: Improvement in participants' knowledge of vaccination safety, benefits, and future uptake intentions.
    2. **Secondary Outcomes**: Identification of barriers to vaccination uptake and socio-demographic factors influencing outcomes.

Questionnaire (Appendix B)

A validated questionnaire will be used in this study to measure vaccine-related knowledge, attitude and future uptake intention. The questionnaire will be adapted from a study conducted by Hong et al in Singapore (7). There are 6 sections in this questionnaire.

Part A: 3 questions for screening purposes to exclude respondents who did not fulfill the inclusion criteria.

Section 1: 9 items on sociodemographic data and the health status of the respondents.

Section 2: 4 items to assess the knowledge on vaccination in pregnancy.

Section 3: 5 items to assess the knowledge about whooping cough.

Section 4: 4 items to assess the knowledge on Influenza Infection.

Section 5: 2 items to assess attitude towards vaccination in general and in pregnancy.

Section 6: 6 items to assess attitude towards vaccination against influenza and whooping cough during pregnancy.

Content and face validity will be conducted among six experts on the questionnaires.

3.6 Data Collection

- **Knowledge and attitude Assessment**: A pre- and post-intervention questionnaire will assess knowledge about the importance, safety, and benefits of vaccination (vaccine literacy)

- **Vaccination future uptake**: Intention of future vaccine uptake of the influenza and Tdap vaccines will be assessed using questionnaire
- **Follow-up**: Participants will be followed up at one month after providing intervention to assess intention of future vaccine uptake and barriers if not intending for vaccination uptake

3.7 Study Duration and Timeline

1st October 2024- 31st December 2026

Proposal preparation: 1 month
Web-based educational module development: 3 months
Application of ethical approval: 3-6 months
Data collection: 12 months

Data analysis: 5 months
Write-up and publication: 3 months

The participation duration for each participant is 2 months

3.8 Study Visits and Procedures

Visit 1: Pre-Data Collection

- Observe clinic workflow to plan and facilitate patient recruitment.
- Administer pre-intervention survey and address any logistical or procedural issues.

Visit 2: Recruitment and Intervention Assignment

- Recruit participants and assign them to either the intervention (InTroDuce-Programme) or standard care arms.

Visit 3: Follow-Up Assessment

- Conduct assessments 1 month post intervention to evaluate post-intervention knowledge, attitudes, and vaccination intention

3.9 Statistical Analysis Plan

Statistical Methods:

a. Primary outcome (knowledge score) will be analyzed using paired t-tests or Mann-Whitney U tests for non-parametric data.
b. Secondary outcomes (vaccination uptake which is yes or no) will be analyzed using multiple logistic regression to determine the factors associated with no vaccination uptake intention.

3.10 Risk and benefit to study participants

Risk: There is minimal risk for participants.

1. Participants may feel uncomfortable answering survey questions about their attitudes or beliefs regarding vaccination and inconvenience due to the time required for surveys. However, surveys will be conducted in a nonjudgmental and private setting, with participants free to withdraw at any time.
2. Participants who choose to get vaccinated as recommended might experience common side effects such as mild fever, soreness at the injection site. However, participants will be informed of possible side effects and how to manage them.

Benefits:

1. Participants in the intervention group will gain valuable knowledge about influenza and Tdap vaccination, potentially increasing their confidence and willingness to vaccinate.

2. By choosing vaccination during the study, participants may lower their own risk of severe influenza or pertussis and protect their newborns.
3. Participants contribute to improving the understanding of barriers and facilitators of vaccination, which could benefit future pregnant women and public health programs.

3.11 Risk Benefit Assessment

As stated above, there is minimal risk from the InTroDuce-Programme (web-based educational module). Study findings shall potentially improve outcomes of participants and future pregnant women. The expected benefit outweighs the minimal risk to participants.

3.12 Ethics of Study

Ethical approval will be obtained from the National Medical Research Registry and Ethics Committee. Permission will be obtained from the Selangor State Health Department and Hulu Langat district office before data collection. Study will be conducted in compliance with ethical principles outlined in the Declaration of Helsinki and Malaysian Good Clinical Practice Guideline.

Pregnant women are included in this study as they are the primary population at risk for influenza and pertussis complications. Vaccination during pregnancy is critical for protecting both the mother and baby from these preventable illnesses. Therefore, their inclusion is essential to understand barriers and improve vaccination uptake in this vulnerable group, which aligns with public health goals.

- Participants will be provided with detailed information about the study, including its purpose, procedures, potential risks, and benefits.
- Written informed consent will be obtained, ensuring they understand their voluntary participation and right to withdraw at any time without penalty or impact on their standard care.
- The intervention involves an educational program, which poses minimal risk. No invasive procedures will be conducted as part of this study.
- Participants with contraindications to vaccination (e.g., severe allergic reactions) will be excluded from the study to ensure safety.

3.13 Informed Consent/Assent Process

Patients shall be informed of the study during their usual clinic visits. If they are willing to participate, the consent forms will be signed and dated. If they need to, they are allowed to take the information sheet home to consult with their family members, and another day for getting consent arranged.

3.14 Privacy and Confidentiality

Participants' names will be kept on a password-protected database and will be linked only with a study identification number for this research. The identification number instead of patient identifiers will be used on participant data sheets. Access to participants' data will be restricted to the study researchers through a password-protected database. The data will be securely stored for a minimum of seven years following the study's completion, after which it will be permanently deleted. Participants will not be allowed to view their personal study data, as the data will be consolidated into a database.

3.15 Conflict of Interest

The investigators declare they have no conflict of interest.

3.16 Publication Policy

No personal information will be disclosed and subjects will not be identified when the findings of the survey are published. Permission from the Director General of Health, Malaysia will be obtained prior to publication.

3.17 Termination of Study

The researchers may decide to terminate the study at any time. Participants will be informed if the study is terminated and follow-up visits will be arranged if needed.

3.18 References

1. Centers for Disease Control and Prevention (CDC). (2023). Vaccination During Pregnancy. [Internet]. Available from: https://www.cdc.gov/vaccines-pregnancy/about/index.html
2. Kilich E, Dada S, Francis MR, Tazare J, Chico RM, Paterson P, et al. Factors that influence vaccination decision-making among pregnant women: A systematic review and meta-analysis. PLoS One. 2020;15(7):e0234827.
3. Deverall EJ, Gilmore B, Illing S, Peiris-John R. Pertussis vaccination uptake in pregnancy: lessons to be learned from an integrated healthcare approach. N Z Med J. 2018 Apr;131(1473):42–7.
4. Razai MS, Mansour R, Goldsmith L, Freeman S, Mason-Apps C, Ravindran P, et al. Interventions to increase vaccination against COVID-19, influenza and pertussis during pregnancy: a systematic review and meta-analysis. J Travel Med. 2023 Dec;30(8).
5. Dawood FS, Kittikraisak W, Patel A, Rentz Hunt D, Suntarattiwong P, Wesley MG, et al. Incidence of influenza during pregnancy and association with pregnancy and perinatal outcomes in three middle-income countries: a multisite prospective longitudinal cohort study. Lancet Infect Dis. 2021;21(1):97–106.
6. Racicot K, Mor G. Risks associated with viral infections during pregnancy. J Clin Invest. 2017 May;127(5):1591–9.
7. Hong EY, Kulkarni K, Gosavi A, Wong HC, Singh K, Kale AS. Assessment of knowledge and attitude towards influenza and pertussis vaccination in pregnancy and factors affecting vaccine uptake rates: a cross-sectional survey. Singapore Med J. 2023 Aug;64(8):513–6.
8. MOH. PERINATAL CARE MANUAL 4th EDITION. Ministry of Health. 2022;(January):1–457.
9. Arora M, Lakshmi R. Vaccines - safety in pregnancy. Best Pract Res Clin Obstet Gynaecol. 2021 Oct;76:23–40.
10. Merdrignac L, Acosta L, Habington A, Garcìa Cenoz M, Pandolfi E, Fabiánová K, et al. Effectiveness of pertussis vaccination in pregnancy to prevent hospitalisation in infants aged <2 months and effectiveness of both primary vaccination and mother's vaccination in pregnancy in infants

aged 2-11 months. Vaccine. 2022 Oct;40(44):6374–82.
11. Razai MS, Mansour R, Ravindran P, Freeman S, Mason-Apps C, Morris J, et al. Facilitators and barriers to vaccination uptake in pregnancy: A qualitative systematic review. PLoS One. 2024;19(4):e0298407.
12. Razai MS, Chaudhry UAR, Doerholt K, Bauld L, Majeed A. Covid-19 vaccination hesitancy. BMJ. 2021 May;373:n1138.
13. Parsons J, Griffiths SE, Thomas N, Atherton H. How effective are digital interventions in increasing flu vaccination among pregnant women? A systematic review and meta-analysis. J Public Health (Oxf). 2022 Dec;44(4):863–76.
14. O'Leary ST, Narwaney KJ, Wagner NM, Kraus CR, Omer SB, Glanz JM. Efficacy of a Web-Based Intervention to Increase Uptake of Maternal Vaccines: An RCT. Am J Prev Med. 2019 Oct 1;57(4):e125–33.
