# Supplementary material for: Effectiveness of the influenza and Tdap vaccination educational module (InTroDuce-Programme) on knowledge and intention for antenatal vaccination: A cluster randomised controlled trial protocol among pregnant women in Malaysian primary care clinics
Source: PLoS One. 2026 Mar 12;21(3):e0344651. doi: 10.1371/journal.pone.0344651 (PMC12981446; doi:10.1371/journal.pone.0344651)
Supplement: S1 Appendix — (DOCX) [file pone.0344651.s002.docx]

**PATIENT INFORMATION SHEET AND INFORMED CONSENT FORM**

*(for adult subjects and interventional studies)*

1. **Title of study**: Effectiveness of the Influenza and Tdap Vaccination Educational and Learning Module (InTroDuce-Programme) in improving knowledge and future uptake of vaccination among pregnant mothers in primary care clinics: A randomised control trial

2. **Name of investigator and institution:**

|  | Investigator | Study site |
| --- | --- | --- |
| 1 | Dr. Lau Hung Chiun (Principal investigator) | Klinik Kesihatan Kajang |
| 2 | Prof Dr. Ching Siew Mooi | Klinik Kesihatan Batu 9 |
| 3 | Dr Nor Hazlin Binti Talib | Klinik Kesihatan Batu 9 |
| 4 | Dr Nur Harnani | Klinik Kesihatan Kajang |
| 5 | Dr Nadiah Binti Md Alwi | Klinik Kesihatan Balakong |
| 6 | Dr Siti Rohani bt. Mohamed Alias | Klinik Kesihatan Bangi |

3. **Name of sponsor**: Universiti Putra Malaysia grant

**4. Introduction:**

You are invited to participate in a research study because you are pregnant that requires *vaccination against influenza and Tdap*. The details of the research trial are described in this document. It is important that you understand why the research is being done and what it will involve. Please take your time to read through and consider this information carefully before you decide if you are willing to participate. Ask the study staff if anything is unclear or if you like more information. After you are properly satisfied that you understand this study, and that you wish to participate, you must sign this informed consent form. To participate in this study, you may be required to provide you doctor with information on your health history; you may harm yourself if you are not truthful with the information provided.

Your participation in this study is voluntary. You do not have to be in this study if you do not want to. You may also refuse to answer any questions you do not want to answer. If you volunteer to be in this study, you may withdraw from it at any time. If you withdraw, any data collected from you up to your withdrawal will still be used for the study. Your refusal to participate or withdrawal will not affect any medical or health benefits to which you are otherwise entitled.

This study has been approved by the Medical Research and Ethics Committee, Ministry of Health Malaysia.

**5. What is the purpose of the study?**

We are conducting this study to see if an online educational program, called the InTroDuce-Programme, can help pregnant mothers better understand the importance of getting vaccinated against influenza (flu), tetanus, diphtheria, and pertussis (whooping cough). Even though these vaccines are proven to be safe and effective, many pregnant women still do not receive them. In Malaysia, there has been little research on how to improve vaccination rates during pregnancy. This study aims to provide useful information on whether a digital education program can help improve knowledge, address concerns and encourage more mothers to get vaccinated.

A total of 351 pregnant women will take part in this study. The entire study will last about 12 months, but your participation will only take about one month.

**6. What kind of intervention will I receive?**

If you agree to participate in the study, the doctor may need to perform some tests and examinations to determine if you are suitable for the study. If you are deemed suitable, you will be randomly (by chance, like flipping a coin) assigned to either intervention group or control group. You have equal chance of being assigned to each of the group.

Group 1: Web-based educational module on influenza and Tdap vaccination via the InTroDuce-Programme.

Group 2: Standard antenatal care (placebo).

**7. What will happen if I decide to take part?**

The study procedures involve a comprehensive approach to examining vaccination knowledge among pregnant women. Participants will first be screened using predefined inclusion and exclusion criteria and complete a pre-intervention questionnaire. They will then be randomly assigned to either the intervention or control group. There is no blinding of the intervention here. The intervention group will receive a web-based educational module about influenza and Tdap vaccination, while the control group will continue to receive standard antenatal care. One month after the intervention, a follow-up assessment will be conducted, culminating in a final questionnaire designed to evaluate changes in participants' knowledge and vaccination intentions.

**8. When will I receive the trial product and how should it be kept?**

For group 1(intervention), you will receive a web-based educational module about influenza and Tdap vaccination after the recruitment. Explanation will be given to the participants in the form of video. The module comprises several videos covering topics such as the purpose of vaccination, how it works, potential side effects, safety in pregnancy, concerns and worries about vaccination.

For group 2 (control), you will continue to receive standard antenatal care. The control group will be given the web-based educational module after one month. You may store the educational materials on your phone or elsewhere.

**9. What are my responsibilities when taking part in this study?**

It is important that you answer the questions honestly and completely. If your condition or circumstances change during the study, you must tell the study doctor. You must not join the intervention without consulting your study doctor. You must inform your study doctor immediately if you make any changes to intervention programme. It is very important that your study doctor be informed very rapidly of any eventual changes to your health during your participation in the study. For your own security, it is important that you follow your study doctor’s instructions throughout the entire duration of the study.

**10. What kind of treatment will I receive after my participation in the trial?**

You will receive standard treatment in the health clinic.

**11. What are the potential risks and side effects of being in this study?**

The risks associated with this study are minimal. However, potential risks and side effects of web-based interventions may include the misinterpretation of information, where patients could misunderstand health-related content, potentially leading to inappropriate medical decisions without professional guidance.

**12. What are the benefits of being in this study?**

The benefits of participating in this study are as follows:

1. Participants will gain comprehensive knowledge about influenza and Tdap vaccination during pregnancy through an educational module.

2. The study provides an opportunity to improve personal understanding of vaccination safety, benefits, and importance.

3. Participants contribute to research that may help improve vaccination uptake rates among pregnant women in Malaysia.

4. The intervention aims to address barriers to vaccination and provide evidence-based information to support informed health decisions.

5. Even if not directly benefiting personally, participants help advance medical knowledge that could improve future maternal and infant health strategies.

6. The participants will be informed regarding their improvement in knowledge score via email at the end of the study.

**13. What if I am injured during this study?**

You should seek evaluation and treatment if you are injured as a result of being in this study. However, in view of this study is an intervention on education and not involving any physical activities or invasive procedure, thus, this section is not relevant to this study. There are no anticipated expenses for this study as we conduct the study during the routine follow-up.

**14. What are my alternatives if I do not participate in this study?**

You do not have to participate in this study to get treatment for your disease or condition. The study doctor will discuss in more details the benefits and risks of those treatments with you.

**15. Who is funding the research?**

This study is sponsored by UPM grant. All other drugs and procedures that are not required by the study but are part of your routine medical care will have to be paid by you or your insurance. The sponsor will financially compensate the time spent by the study staff, use of facilities, etc., for including you in the study. You will be reimbursed RM 20 at baseline and RM10 on subsequent visit.

**16. Can the research or my participation be terminated early?**

The study doctor or the sponsor may be due to concerns for your safety, stop the study or your participation at any time. If the study is stopped early for any reason, you will be informed and arrangements made for your future care. You may be asked to attend a final follow-up visit.

**17. Will my medical information be kept private?**

All your information obtained in this study will be kept and handled in a confidential manner, in accordance with applicable laws and regulations. When publishing or presenting the study results, your identity will not be revealed without your expressed consent. Individuals involved in this study and in your medical care and governmental or regulatory authorities may inspect your medical records, where appropriate and necessary. Data from the study may be archived for the purpose of analysis, but your identity will not be revealed at any time.

**18. Who should I call if I have questions?**

If you have any questions about the research, please contact the study doctor Dr Ivy Lau Hung Chiun (016 3502549) or Prof Dr Ching Siew Mooi (0193806652). If you have any questions about your rights as a participant in this study, please contact: The Secretary, Medical Research & Ethics Committee, Ministry of Health Malaysia, at telephone number 03-3362 8407 / 8205 / 8888.

**INFORMED CONSENT FORM**

Title of Study: Effectiveness of the Influenza and Tdap Vaccination Educational and Learning Module (InTroDuce-Programme) in improving knowledge and future uptake of vaccination among pregnant mothersin primary care clinics: A randomised control trial

By signing below I confirm the following:


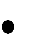
 I have been given oral and written information for the above study and have read and understood the information given.


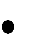
 I have had sufficient time to consider participation in the study and have had the opportunity to ask questions and all my questions have been answered satisfactorily.


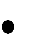
 I understand that my participation is voluntary and I can at anytime free withdraw from the study without giving a reason and this will in no way affect my future treatment. I am not taking part in any other research study at this time. I understand the risks and benefits, and I freely give my informed consent to participate under the conditions stated. I understand that I must follow the study doctor’s (investigator’s) instructions related to my participation in the study.


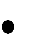
 I understand that study staff, qualified monitors and auditors, the sponsor or its affiliates, and governmental or regulatory authorities, have direct access to my medical record in order to make sure that the study is conducted correctly and the data are recorded correctly. All personal details will be treated as STRICTLY CONFIDENTIAL

- You will be informed of any new information made to the current Patient Information Sheet/ Informed Consent Form (PIS/ICF).

**Subject:**

| Signature: | I/C number: |
| --- | --- |
| Name: | Date: |

**Investigator conducting informed consent:**

| Signature: | I/C number: |
| --- | --- |
| Name: | Date: |

**Impartial witness:** *(Required if subject is illiterate and contents of patient information sheet is orally communicated to subject)*

| Signature: | I/C number: |
| --- | --- |
| Name: | Date: |
